# Concerted *In Vitro* Trimming of Viral HLA-B27-Restricted Ligands by Human ERAP1 and ERAP2 Aminopeptidases

**DOI:** 10.1371/journal.pone.0079596

**Published:** 2013-11-01

**Authors:** Elena Lorente, Alejandro Barriga, Carolina Johnstone, Carmen Mir, Mercedes Jiménez, Daniel López

**Affiliations:** Centro Nacional de Microbiología, Instituto de Salud Carlos III, Madrid, Spain; Massachusetts General Hospital, United States of America

## Abstract

In the classical human leukocyte antigen (HLA) class I antigen processing and presentation pathway, the antigenic peptides are generated from viral proteins by multiple proteolytic cleavages of the proteasome (and in some cases other cytosolic proteases) and transported to the endoplasmic reticulum (ER) lumen where they are exposed to aminopeptidase activity. In human cells, two different ER-resident enzymes, ERAP1 and ERAP2, can trim the N-terminally extended residues of peptide precursors. In this study, the possible cooperative effect of generating five naturally processed HLA-B27 ligands by both proteases was analyzed. We identified differences in the products obtained with increased detection of natural HLA-B27 ligands by comparing double versus single enzyme digestions by mass spectrometry analysis. These *in vitro* data suggest that each enzyme can use the degradation products of the other as a substrate for new N-terminal trimming, indicating concerted aminoproteolytic activity of ERAP 1 and ERAP2.

## Introduction

Newly synthesized viral proteins are proteolytically processed, mainly by the action of proteasomes [1] and in some cases of other cytosolic proteases [2]. This protein degradation generates an extremely diverse pool of substrates, both in sequence and length, that are translocated to the lumen of the endoplasmic reticulum (ER) by transporters associated with antigen processing. Among them, only a small fraction with a correct epitope or NH_2_-terminally-extended precursors can be used for antigen presentation by direct epitope binding to HLA class I molecules or by precursor editing and customization, respectively, to yield the final viral epitope by ER-resident aminopeptidase activity. Later, binding of a viral peptide to HLA class I molecules in the ER stabilizes the nascent antigenic complexes and allows for their subsequent transport to the cell membrane, where they are exposed to antiviral CD8^+^ cytotoxic T lymphocyte (CTL) activity that recognizes and kills virus-infected cells [3]. 

The ER-resident enzymatic activity that trims N-terminally extended residues of peptide precursors to their final length has been identified as the ER aminopeptidase associated with antigen processing (ERAAP) in mice [4] and ER aminopeptidase 1 (ERAP1) in humans [5,6]. Additionally, a second ER aminopeptidase, ERAP2, also trims certain precursors to HLA class I-presented antigenic peptides in humans but not in mice [7,8]. The homology between the two human enzymes is less (approximately 50% identity) compared to the human ERAP1 and mouse ERAAP homologs, according to their non-redundant trimming activities. ERAP1 and ERAP2 form complexes in low amounts [9], and a complementary and concerted function of these human trimming aminopeptidases in HLA class I peptide presentation with a single substrate has been previously described using a 15-mer peptide, derived from the HIV IIIB envelope protein. Six N-terminal extended residues regarding the shortest natural 9-mer epitope presented by the H-2L^d^-restricted CTL response in BALB/C mice [10], were cooperatively trimmed *in vitro* to generate the 9-mer epitope by concerted activity of these two aminopeptidases [9]. Therefore, is this case of a rare N-terminal cooperative trimming xenogeneic event? Or, conversely, do ERAP1 and ERAP2 show a broad capacity to cooperatively generate multiple HLA class I viral epitopes? To answer these questions, the possible cooperative effect of both of these metallopeptidases was analyzed using N-terminal extended precursors of five naturally processed HLA-B27 ligands, 9, 10, and 11 amino acids in length, derived from human respiratory syncytial virus (HRSV) [11]. This *in vitro* study demonstrates differences in the products obtained from single digestions when compared with the mixture of both enzymes, indicating a concerted action of ERAP1 and ERAP2 aminopeptidases.

## Materials and Methods

### Synthetic peptides

**Figure 1:**
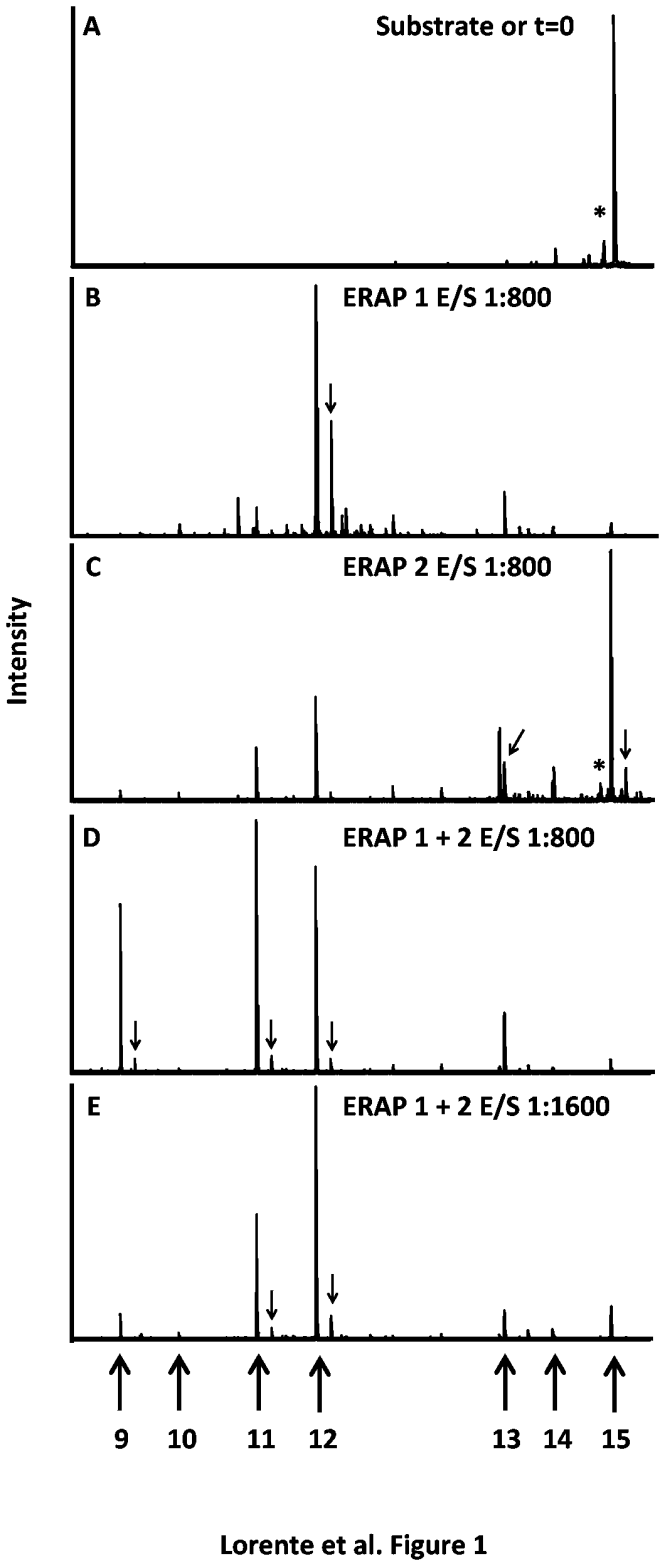
MALDI-MS analysis of the M_163-177_ synthetic peptide digested with purified ERAPs. M_163-177_ substrate (Panel A) was digested overnight with ERAP as follow: ERAP1 at an E/S ratio of 1:800 (panel B), ERAP2 at an E/S ratio of 1:800 (panel C), both ERAP1 and 2 at an E/S ratio of 1:800 for each enzyme (panel D), and both ERAP1 and 2 at an E/S ratio of 1:1600 for each enzyme (panel E). MALDI-TOF analysis of digestions detected M_163-177_ substrate and N-trimmed peptides, as well as several adducts and neutral loss of peptides (marked by an arrow or asterisk, respectively). The *m*/*z* range represented in the *x*-axis is 1000-1850. The *m*/*z* position and length of each possible N-trimmed peptide is indicated with an arrow at the base of the figure.

Peptides were synthesized in a peptide synthesizer (model 433A; Applied Biosystems, Foster City, CA) and purified by RP-HPLC (purity > 99%, for an example see Figure 1A). Identities were confirmed by matrix-assisted laser desorption/ionization time-of-flight (MALDI-TOF) mass spectrometry. 

### Digestion with recombinant ERAP enzymes

Recombinant purified human ERAP1^181Q528R^ and ERAP2^392K^ enzymes (R&D Systems, Minneapolis, MN, USA) were incubated for the indicated periods at 37°C with purified synthetic peptides at the indicated molar enzyme/substrate ratios in 25 mM Tris and pH 8. The addition of trifluoroacetic acid to a final concentration of 0.2% stopped the digestions and denatured the proteins. In the experiments, different E/S ratios (1:400, 1:800, and 1:2,000) were used with similar results, thus the 1:800 E/S ratio was selected for graphic purposes.

### MALDI-TOF mass spectrometry

MALDI-TOF mass spectrometry was performed using a Reflex IV instrument (Brucker-Franzen Analytik, Bremen, Germany) operating in the positive ion reflection mode. One μl of sample was mixed with 0,8 μl of saturated α-cyanohydroxycinnamic acid matrix in the same solution. One microliter of the mixture was dried and subjected to analysis as previously described [12].

 The intensity peaks obtained at each time point for all peptides were added and taken as 100% for each time point and depicted as stacked area charts. Data shown are representative of 2-4 different experiments. To show both sensitivity and specificity of mass spectrometry analysis, a calibration curve of three nested set synthetic peptides is depicted in Figure S1.

## Results

### Two natural HRSV 9-mer ligands are efficiently generated by concerted ERAP1 and ERAP2 aminopeptidase activity

To study susceptibility to aminopeptidase activity, recombinant ERAP1 was incubated with several 15-mer synthetic peptide precursors of naturally processed viral HLA-B27 ligands from HRSV-infected cells described previously [11]. Cleavage products generated at different enzymatic digestion times were subsequently analyzed by mass spectrometry. At time 0 or in the absence of enzyme, this system allowed the detection of molecular species with the same monoisotopic *m*/*z* as the 15-mer substrate (an example with the M_163-177_ synthetic peptide is shown in Figure 1A). Additionally, minority peaks with a 15-mer *m*/*z* less than 16 or 36 Daltons were frequently found (Figure 1A, asterisk), probably due to the previously reported neutral loss in the peptides under desorption/ionization conditions [12]. After overnight incubation in the presence of the enzyme, total cleavage of the M_163-177_ substrate was observed via detection of trimmed 12-mer and 13-mer products (Figure 1B). Additionally, trimmed peptide adducts were found in some experiments (Figure 1, arrows), as previously reported in similar desorption/ionization conditions [12].

**Figure 2:**
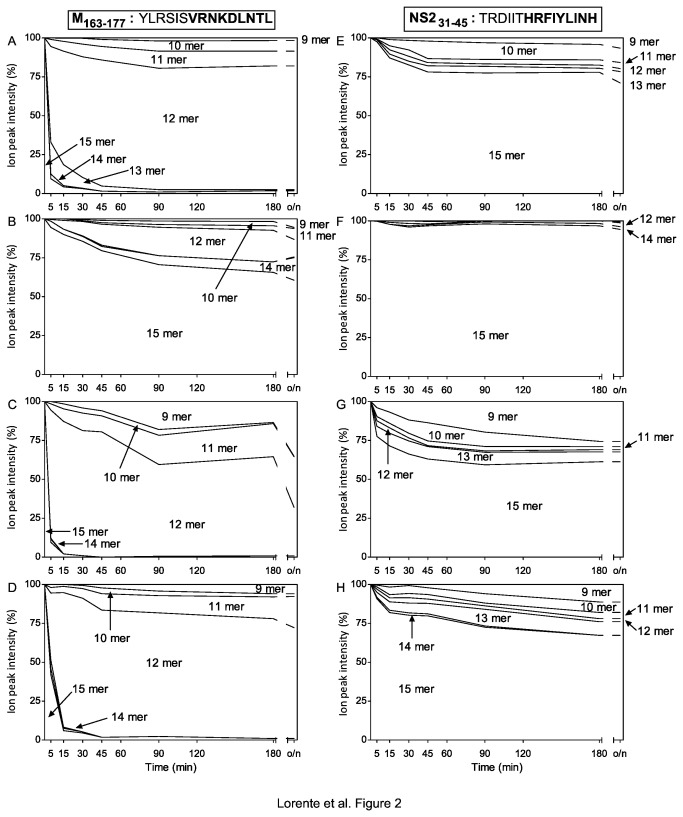
Stacked area charts of M_163-177_ and NS2_31-45_ synthetic peptides digested with purified ERAPs. M_163-177_ (panels A-D) and NS2_31-45_ (panels E-H) (sequences are indicated at the top of the figure and the respective HLA ligands identified by MS are bolded) synthetic peptides were digested at different times with ERAP enzymes as follows: ERAP1 at an E/S ratio of 1:800 (panels A and E), ERAP2 at an E/S ratio of 1:800 (panels B and F), both ERAP1 and 2 at an E/S ratio of 1:800 (panels C and G), and both ERAP1 and 2 at an E/S ratio of 1:1600 (panels D and H). The intensity peaks obtained by MALDI-TOF analysis for all peptides at each time point were added and taken as 100% for each time point and depicted. The different N-end trimming products detected are named in their respective region. The results depicted are the mean values of three or four independent experiments.

The results shown in Figure 1 and other intermediate points are summarized and depicted in Figure 2A for further clarification. After only 5 minutes in the presence of the enzyme, major substrate cleavage was observed and trimmed 14-mer, 13-mer, and 11-mer products with a predominant 12-mer signal were detected (Figure 2A). After 15 minutes, traces of the 10-mer peptide were also found. After 45 minutes, the signal of the 9-mer, which is the natural ligand identified in HRSV-infected cells, was detected. Peptides shorter than the 9-mer were not detected in the experiments. 

Next, similar experiments with this 15-mer synthetic peptide precursor and ERAP2 enzyme were carried out in parallel. Predominantly untrimmed peptides, following an overnight incubation, were detected with M_163-177_ substrate (Figure 2B). Low levels of several products, including both 9-mers, were detected in this experiment (Figure 2B).

To test the complementary trimming function of both aminopeptidases, similar experiments with five 15-mer synthetic peptide precursors and a mix of both enzymes were carried out in parallel to individual digestions. Figure 2C shows the stacked area charts of M_163-177_ synthetic peptide digested with the mix of both aminoproteases. The 15-mer M_163-177_ peptide was more efficiently trimmed to both the short 11-mer product and especially the natural 9-mer ligand (Table 1) in the presence of both enzymes (Figure 1D and stacked area charts 2C) than in individual digestions (Figures 1B and 2C, 2A and 2B). Additionally, when the E/S ratio decreased by half, the 11-mer product was generated more in the enzyme mix than in individual digestions (Figure 2D versus Figure 2A and 2B), similar to the production of the natural 9-mer ligand (6% of total with the mix versus 7% with the sum of individual digestions), as shown in Table 1. 

**Table 1:** Summary of HRSV HLA-B27 ligands or shorter peptides generated by ERAP enzymes.

Name	Substrate	Ligand ^a^	Percentage of detected product after overnight incubation				mer ^e^
			ERAP 1 ^b^	ERAP 2 ^b^	ERAP 1+2 ^c^	ERAP 1+2 ½ ^d^	
M _163-177_	YLRSISVRNKDLNTL	VRNKDLNTL	2 ^f^	5	35	6	9
NS2 _31-45_	TRDIITHRFIYLINH	HRFIYLINH	7	0	26	11	9
N _191-205_	KNEMKRYKGLLPKDI	KRYKGLLPKDI	25	2	12	23	11
			36	7	5	8	10
			8	6	47	25	9
P _194-208_	AMARLRNEESEKMAK	LRNEESEKMAK	4	21	44	38	11
			0	3	3	5	10
N _95-109_	VDVTTHRQDINGKEM	HRQDINGKEM	0	0	0	0	10

^a^ Ligand detected by mass spectrometry analysis in HRSV-infected cells [11].

^b^ E/S molar ratio 1:800

^c^ E/S molar ratio 1:800 with each enzyme.

^d^ E/S molar ratio 1:1,600 with each enzyme.

^e^ Length of detected product.

^f^ Percentage of detected product after overnight incubation.

Similar experiments were performed with the 15-mer NS2_31-45_ synthetic peptide. This substrate was relatively resistant to ERAP1 activity because low amounts of several N-trimmed products were detected, even after long enzymatic reaction times (Figure 2E); however, amounts of 9-mer natural ligand were detected (Table 1). Also, ERAP2 was incapable of trimming the NS2_31-45_ synthetic peptide, as only low amounts of the 14-mer and 12-mer products were detected at all analyzed times (Figure 2F).

Similarly to M_163-177_ peptide, the NS2_31-45_ peptide experiments with a mix of both ERAP enzymes demonstrated increased NS2_31-45_ precursor destruction and trimming rates to shorter peptides (Figure 2G versus Figure 2E and 2F and Table 1). This observation held true even when the E/S ratio was decreased by half: 11% of the 9-mer natural ligand was produced in the enzyme mix condition versus 7% with ERAP1 (Table 1). These data show that the combined activity of both enzymes improves the *in vitro* generation efficiency of both natural HLA-B27 ligands. 

### Differential role of combined ERAP1 and ERAP2 activity in the natural HRSV 11-mer ligand generation

**Figure 3:**
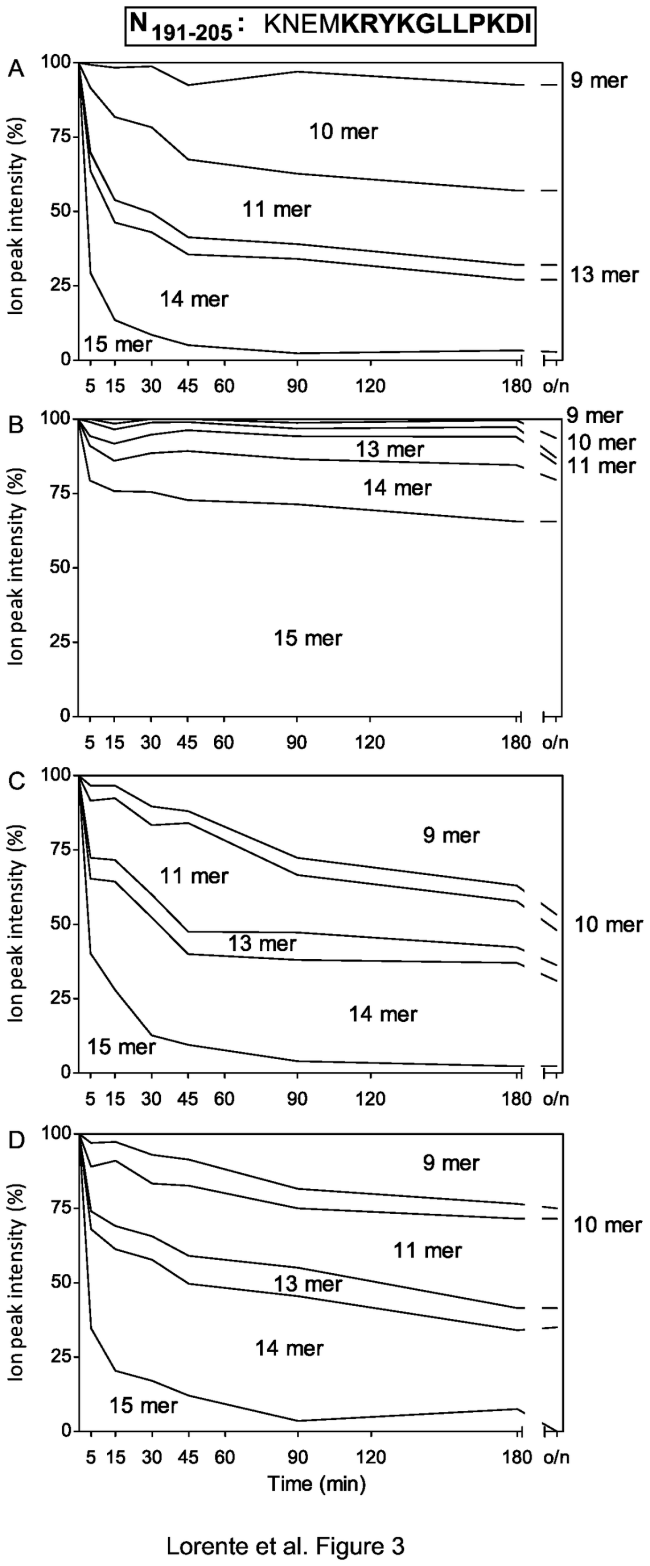
Stacked area charts of the N_191-205_ synthetic peptide digested with purified ERAPs. The N_191-205_ synthetic peptide (sequence indicated at the top of the figure and the 11-mer identified by MS is bolded) was digested at different times with ERAP enzymes as follows: ERAP1 at an E/S ratio of 1:800 (panel A), ERAP2 at an E/S ratio of 1:800 (panel B), both ERAP1 and 2 at an E/S ratio of 1:800 (panel C), and both ERAP1 and 2 at an E/S ratio of 1:1600 (panel D). The results depicted as Figure 2 are the mean values of three or four independent experiments.

Similar to the M_163-177_ synthetic peptide, the incubation of 15-mer N_191-205_ precursor with ERAP1 predominantly cleaved the substrate and different N-trimmed products were detected, although without a major product (Figure 3A), and low levels of several products, including both 9-mers, were detected in the experiment with ERAP2 (Figure 3B). The activity of ERAP1 (Figure 3A) but not ERAP2 (Figure 3B) over N_191-205_ synthetic precursor efficiently generated the previously identified by mass spectrometry analysis natural 11mer ligand in addition to other trimmed products (Table 1). 

Furthermore, the comparison between single versus double enzymatic source trimming of this N_191-205_ synthetic peptide revealed a strong reduction in the amounts of both the natural 11-mer ligand (from 25% to 12% after overnight incubation) and the 10-mer, whereas there was an increase in the 9-mer product (Figure 3C and Table 1). When an E/S ratio of half was used, 11-mer product generation was restored by 25% with ERAP1 versus 23% with the mix of both enzymes (Figure 3D and Table 1). These data indicate that both aminopeptidases exhibit concerted non-destructive proteolytic activity only at lower concentrations. 

### Generation without destruction of a natural HRSV 11-mer ligand by concerted ERAP1 and ERAP2 aminopeptidase peptide trimming

**Figure 4:**
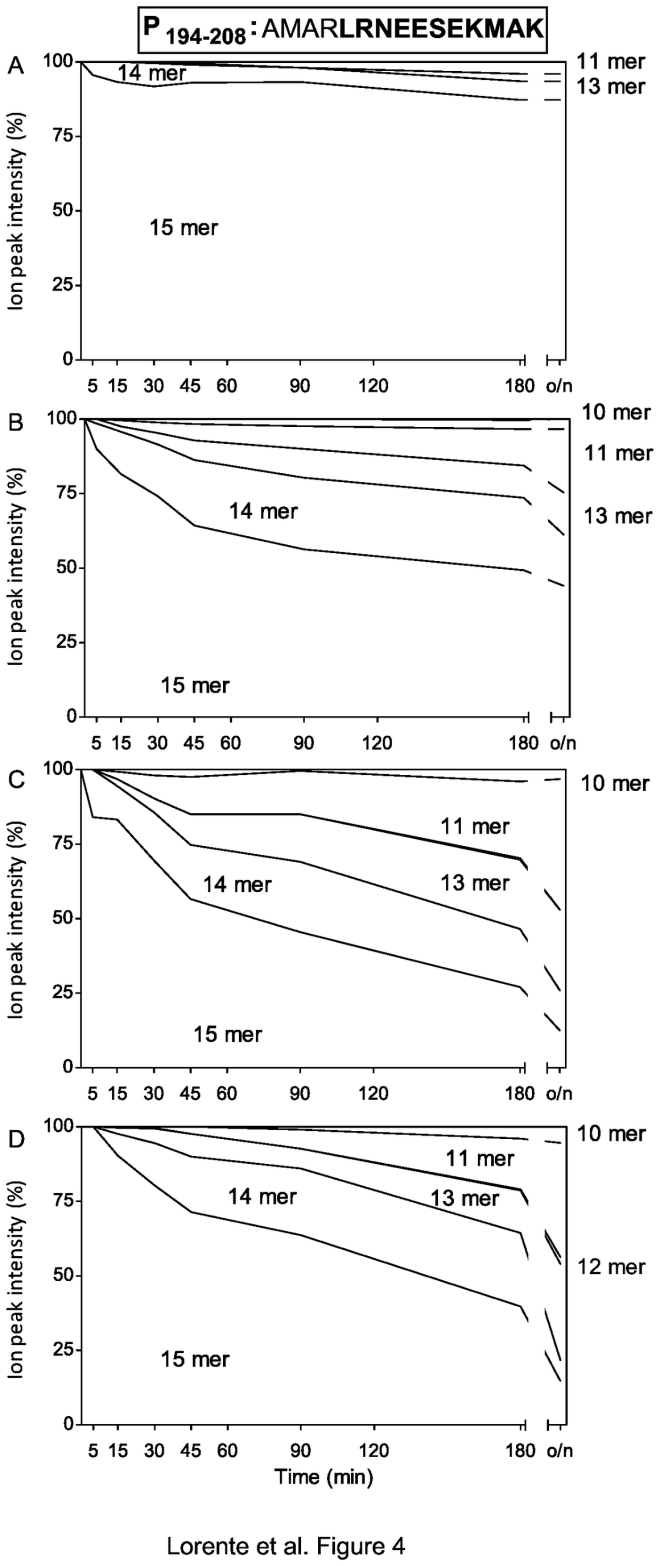
Stacked area charts of the P_194-208_ synthetic peptide digested with purified ERAPs. The P_194-208_ synthetic peptide (sequence indicated at the top of the figure and the 11-mer identified by MS is bolded) was digested at different times with ERAP enzymes as follows: ERAP1 at an E/S ratio of 1:800 (panel A), ERAP2 at an E/S ratio of 1:800 (panel B), both ERAP1 and 2 at an E/S ratio of 1:800 (panel C), and both ERAP1 and 2 at an E/S ratio of 1:1600 (panel D). The results depicted as Figure 2 are the mean values of three or four independent experiments.

The P_194-208_ precursor was marginally trimmed by ERAP1 (Figure 4A). In contrast, several N-trimmed products (including the natural 11-mer ligand) were generated by ERAP2 activity (Figure 4B). This N-extended precursor was processed efficiently to various short products, especially the natural 11-mer ligand, in the presence of both enzymes at both E/S ratios utilized without any epitope destruction, as evidenced by detecting only 3-5% of the 10-mer peptide (Figure 4C and 4D and Table 1). Thus, these results indicate concerted peptide trimming by human ERAP1 and ERAP2 aminopeptidases to generate a long natural ligand.

### ERAP1 and ERAP2 play no role in the natural HRSV 10-mer ligand production

**Figure 5:**
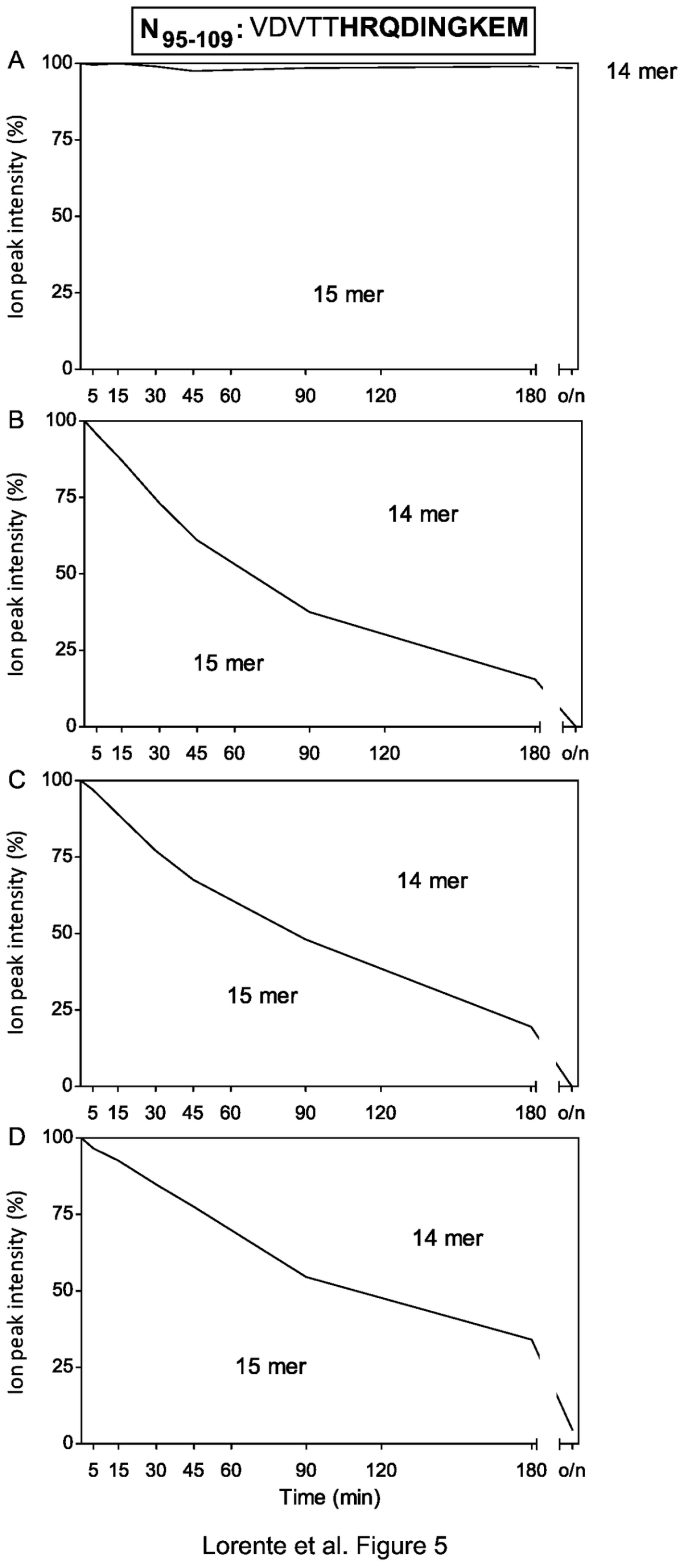
Stacked area charts of the N_95-109_ synthetic peptide digested with purified ERAPs. The N_95-109_ synthetic peptide (sequence indicated at the top of the figure and the 9-mer identified by MS is bolded), was digested at different times with ERAP as follows: ERAP1 at an E/S ratio of 1:200 (panel A), ERAP2 at an E/S ratio of 1:200 (panel B), both ERAP1 and 2 at an E/S ratio of 1:200 (panel C), and both ERAP1 and 2 at an E/S ratio of 1:400 (panel D). The results depicted as Figure 2 are the mean values of two independent experiments.

The N_95-109_ synthetic peptide was not trimmed by ERAP1, and only low amounts of the 14-mer product were detected after ERAP2 activity at the same E/S ratio used in the four previous 15-mer substrates (data not shown). To confirm this point, enzymatic reactions with larger amounts of ERAP enzymes were performed. A pattern was observed similar to the one shown in previous experiments. The N_95-109_ substrate was resistant to the ERAP1 enzyme (Figure 5A). Only one difference was found with ERAP2: the substrate was trimmed to the 14-mer product without detection of shorter peptides (Figure 5B). The mix of both enzymes at both E/S ratios generated an identical pattern (Figure 5C and 5D). Therefore, some peptides, such as N_95-109_, are resistant to individual ERAP1 and ERAP2 aminopeptidases as well as to their mixture. 

## Discussion

This study was undertaken to analyze both ERAP1 and ERAP2 activity and the possible cooperative effects of these ER aminopeptidases in the antigen processing of several HRSV ligands. First, we used mass spectrometry to identify that ERAP1 and/or ERAP2 N-terminal trimming activities yielded the respective natural ligands in four of the five HLA-B27-restricted precursors used. Thus, if these data were typical of the ER-resident trimming, most precursor peptides could be substrates for these aminopeptidases, with only a low fraction of peptides resisting this HLA class I customization. 

Our results indicate that cooperative peptide trimming of M_163-177_ and NS2_31-45_ precursors by ERAP1 and ERAP2 predominantly generated 9-mer peptides as well as the 11-mer ligand from the P_194-208_ substrate (and to a lesser extent from the N_191-205_ peptide at a low E/S ratio) without destroying the natural HLA class I ligands. In contrast to many HLA class I alleles that usually bind peptides 8-10 amino acids in length (SYFPEITHI database: http://www.syfpeithi.de [13]), HLA-B27 could accommodate peptides up to 13 residues in a bulged conformation (SYFPEITHI database and [14]). Thus, the previously reported ERAPs’ N-terminal trimming activity to yield mainly octamer and nonamer products suggested a predominant destructive role of these enzymes in the HLA-B27-restricted ligand antigen processing. The data shown in the present study indicate that these aminopeptidases could be relevant in generating extended peptides without destroying naturally present long ligands or epitopes.

Concerted peptide trimming by human ERAP1 and ERAP2 aminopeptidases was previously described with a single substrate [9]. Subsequently, the molecular basis for the ERAP1-ERAP2 heterodimer formation has been speculated [15]. Our *in vitro* results with several precursors of the naturally processed HLA-B27-restricted ligands from human cells that express both aminopeptidases in their ER lumen indicate that the HIV envelope protein epitope that is presented by murine MHC is not a single and rare N-terminal trimming xenogeneic event generated by the cooperative effect of both enzymes in the antigen processing of MHC class I ligands and reinforce the hypothesis that ERAP1 and ERAP2 have evolved to perform joint actions modulating the repertoire of ligands and epitopes presented by the HLA class I molecules in human cells. 

In addition, similar to our present report, a concerted endoproteolytic activity by caspase 5 and 10 proteases was required for the digestion of a 19-mer peptide with two amino- and carboxy-terminal extensions of five residues [16] to generate a 9mer peptide, which is a natural epitope endogenously processed from murine cytomegalovirus pp69 phosphoprotein [17]. Thus, the concerted activity of related and even unrelated [18,19] enzymes may not be a rare event in antigen processing of specific epitopes and its relevance must be evaluated in future studies with other epitopes.

Our results indicating *in vitro* cooperative peptide trimming by ERAPs enzymes must be expanded in future analyzing the antiviral CD8^+^ T cell responses against these HRSV ligands from human infected-individuals using knocking or silenced cells in each individual aminopeptidase to describe the *in vivo* relevance of the concerted trimming phenomenon by ERAP1 and ERAP2.

## Supporting Information

Figure S1 **Stacked area charts of mixtures of 15-mer, 12-mer and 9-mer synthetic peptides corresponding to the M_163-177_ precursor.**
(EPS)
